# A twenty-four-hour observational study of hand hygiene compliance among health-care workers in Debre Berhan referral hospital, Ethiopia

**DOI:** 10.1186/s13756-017-0268-y

**Published:** 2017-10-30

**Authors:** Tufa Kolola, Takele Gezahegn

**Affiliations:** Department of public health, Debre Berhan University, P.O.Box 445, Debre Berhan, Ethiopia

**Keywords:** Hand hygiene compliance, Direct observation method, Health-care worker, Debre Berhan, World health organization

## Abstract

**Background:**

Hand hygiene (HH) is recognized as the single most effective strategy for preventing health care–associated infections. In developing countries, data on hand hygiene compliance is available only for few health-care facilities. This study aimed to assess hand hygiene compliance among health-care workers in Debre Berhan referral hospital, Ethiopia.

**Methods:**

This study employed the WHO hand hygiene observation method. Direct observation of the health care workers (HCWs) was conducted using an observation record form in five different wards. Trained and validated observers watched HCWs while they had direct contact with patients or their surroundings, and the observers then recorded all possible hand hygiene opportunities and hand hygiene actions. Observation was conducted over a 24 h period to minimize selection bias. More than 200 opportunities per ward were observed according to WHO recommendation, except in neonatal intensive care unit. HH compliance was calculated by dividing the number of times hand hygiene was performed by the total number of opportunities for hand hygiene. A 95% confidence interval (CI) was computed for compliance with the exact binomial method.

**Results:**

A total of 917 hand hygiene opportunities were observed during the study. Overall HH compliance was 22.0% (95% CI: 19.4–24.9). HH compliance was similar across all professional categories and did not vary by shift. Levels of compliance were lower before patient contact (2.4%; 95% CI: 0.9–5.3), before an aseptic procedure (3.6%; 95% CI: 1.6–7.6) and after contact with patient surroundings (3.3%; 95% CI: 1.2–7.9), whereas better levels of compliance were found after body fluid exposure (75.8%; 95% CI: 68.0–82.3) and after patient contact (42.8%; 95% CI: 35.2–50.7).

**Conclusion:**

HH compliance of HCWs was found to be low in Debre Berhan referral hospital. Compliance with indications that protect patients from infection was lower than that protect the HCWs. The findings of this study indicate that HH compliance needs further improvement.

**Electronic supplementary material:**

The online version of this article (doi:10.1186/s13756-017-0268-y) contains supplementary material, which is available to authorized users.

## Background

Health care-associated infections (HAIs) are a major threat to patient safety worldwide [1–4]. Such infections spread between patients in the health-care settings by various means, mainly via the hands of health-care workers (HCWs) [5, 6].

Hand hygiene (HH) is the single most effective strategy for preventing HAIs [7–9]. Hand hygiene is defined as either rubbing the hands with an alcohol-based handrub or handwashing with soap and water [6]. WHO has launched a multimodal hand hygiene improvement strategy to optimize hand hygiene in health care settings [10]. This strategy is now recommended as the most reliable and evidence-based method for ensuring sustainable hand hygiene improvement around the world [11–16]. HCWs compliance with hand hygiene during routine patient care is an integral part of this strategy. HH compliance is measured in a variety of ways. These include: direct observation, handrub consumption, and survey methods [17, 18]. Direct observation of HCWs using WHO’s hand hygiene observation tool is currently recognized as the gold standard for hand hygiene monitoring in the sequence of care [6, 10, 17].

World Health Organization has endorsed “My five moments for hand hygiene” approach, the moments when hand hygiene is required, to effectively interrupt the spread of HAIs [19]. This approach encourages HCWs to clean their hands, i.e., (1) before patient contact, (2) before an aseptic procedure, (3) after body fluid exposure, (4) after patient contact and (5) after contact with patient surroundings [20]. The World Health Organization (WHO) has defined these moments as hand hygiene opportunities (HHOs) to which HCWs should comply with [10, 18]. Hand hygiene opportunity exists whenever one of the indications for hand hygiene occurs. Each opportunity corresponds to hand hygiene action [18].

In developing countries with high burden of health-care-associated infections, improving HCWs compliance with hand hygiene during routine patient care is urgently needed for the patient safety [2, 21]. Despite the clear benefits of hand hygiene practices in healthcare settings, compliance remains an issue in developing countries [11]. In Ethiopia, data on hand hygiene compliance is available only for few health-care facilities [14, 22]. In Debre Berhan referral hospital, HCWs compliance to the WHO’s five moments for hand hygiene was not investigated so far. This study aimed to assess hand hygiene compliance among health-care workers in Debre Berhan referral hospital through direct observation of the WHO’s five moments. The result of this study provides insights about hand hygiene compliance level of health care providers.

## Methods

### Study setting

A cross-sectional study was conducted in Debre Berhan referral hospital using the WHO hand hygiene observation method. Debre Berhan referral hospital is located in North Shoa Zone of Amhara Region which is about 130 km away from Addis Ababa to Dessie. Currently, this hospital serves as a referral centre for a population of North Shoa Zone of Amhara region and for other population from the neighbouring regions. The hospital has a total of 307 HCWs: 38 physicians, 153 nurses, 26 midwives, 7 anaesthetists, 31 laboratory technicians, 2 physiotherapists, 4 dentists, 6 radiographers, 4 optometrists and 36 pharmacists. In addition, 48 medical interns were affiliated to this hospital during data collection. This study was conducted from May 2 to 9, 2017 in the selected wards (Medical, Surgical, Paediatric, Obstetrics and gynecology, and Neonatal intensive care unit) of the hospital. All HCWs, including medical interns, having direct contact with patients or their surroundings in the selected wards were observed.

### Data collection

Data were collected using standardized WHO’s hand hygiene observation tool for direct observation (Additional file 1). Before conducting observation sessions, observers were trained in accordance with the WHO’s hand hygiene observation method [23]. Thereafter, observers were validated by one of the authors based on Sax et al.’s [6] recommendation. In the first case, each observer engaged in an observation session during a patient care situation. Each observer completed the observation form separately while observing the same HCW and the same care sequence. Results were then compared and discordant notifications were discussed. This process was repeated until concordance is reached in terms of the number of hand hygiene opportunities and hand hygiene actions that occurred [6, 20].

In brief, three nurses directly watched 261 HCWs having direct contact with patients or their surroundings, and recorded all possible HHOs and HHAs. Observation was conducted over a 24 h period in each ward to minimize selection bias. The HCWs were unaware of being observed to minimize “Hawthorne effect”. Each HCW was observed for a maximum of four HHOs during the observed care sequence. More than 200 opportunities per ward were observed according to WHO recommendation [23], except in neonatal intensive care unit (NICU). Few opportunities were observed in NICU due to the small number of HCWs working in this unit.

### Data analysis

Data analysis was done using Epi Info 7 and SPSS version 21. Data set underlying the findings is available within the supplementary information files (Additional file 2). Overall compliance was calculated by dividing the number of times hand hygiene was performed by the total number of opportunities for hand hygiene. We also estimated HH compliance by professional categories, and “my five moments for hand hygiene”. A 95% confidence interval (CI) was computed for compliance with the exact binomial method. Overlapping 95% confidence intervals were interpreted as not being significantly different.

## Results

### Hand hygiene compliance

A total of 917 opportunities for hand hygiene were observed during the study. The overall HH compliance was 22.0% (95% CI: 19.4–24.9). HH compliance was 20.6% (95% CI:16.2–25.9) for doctors, 22.9% (95% CI:19.2–27.0) for nurses, 21.2% (95% CI:13.9–30.8) for midwives, and 23.2% (95% CI: 13.4–36.7) for other HCWs. HH compliance was slightly higher in the neonatal intensive care unit (NICU) and paediatric ward compared to other wards. HH compliance varied according to the five moments for hand hygiene. Levels of compliance were lower before patient contact (2.4%%; 95% CI: 0.9–5.3), before an aseptic procedure (3.6%; 95% CI: 1.6–7.6) and after contact with patient surroundings (3.3%; 95% CI: 1.2–7.9). Better levels of compliance were found after body fluid exposure (75.8%; 95% CI: 68.0–82.3) and after patient contact (42.8%; 95% CI: 35.2–50.7) (Table 1).

**Table 1:** Hand hygiene compliance of HCWs in Debre Berhan Referral Hospital, May 2017

Characteristic	Hand hygiene opportunities (n)	Hand hygiene actions (n^a^)	Compliance, % (95% CI)
Over all	917	202	22.0 (19.4–24.9)
Professional category			
Doctor	286	59	20.6 (16.2–25.9)
Nurse	476	109	22.9 (19.2–27.0)
Midwife	99	21	21.2 (13.9–30.8)
Other HCWs	56	13	23.2 (13.4–36.7)
Ward			
Medical	207	44	21.2 (16.0–27.6)
Surgical	203	38	18.7 (13.7–24.9)
Paediatric	211	56	26.5 (20.8–33.1)
OB/GYN	219	43	19.6 (14.7–25.7)
NICU	77	21	27.3 (18.0–38.8)
Shift			
Morning	369	86	23.3 (19.2–28.0)
After noon	318	74	23.3 (18.8–28.4)
Night	230	52	22.6 (17.5–28.4)
Indications			
bef. Pat	255	6	2.4 (0.9–5.3)
bef. Asept	195	7	3.6 (1.6–7.6)
aft.b.f	149	113	75.8 (68.0–82.3)
aft.pat	166	71	42.8 (35.2–50.7)
aft.p.surr	152	5	3.3 (1.2–7.9)

*bef. Pat* before patient contact, *bef. Asept* before an aseptic procedure, *aft.b.f* after body fluid exposure, *aft.pat* after patient contact, *aft.p.surr* after contact with patient surroundings, *HCWs* Health care workers, *n* Number of opportunities for hand hygiene, *n* ^*a*^ Number of positive hand hygiene actions, *CI* Confidence interval, *Other HCWs*, Laboratory technician, dentist and physiotherapist, *NICU* Neonatal intensive care unit, *OB/GYN*, Obstetrics and gynecology

**Fig. 1:**
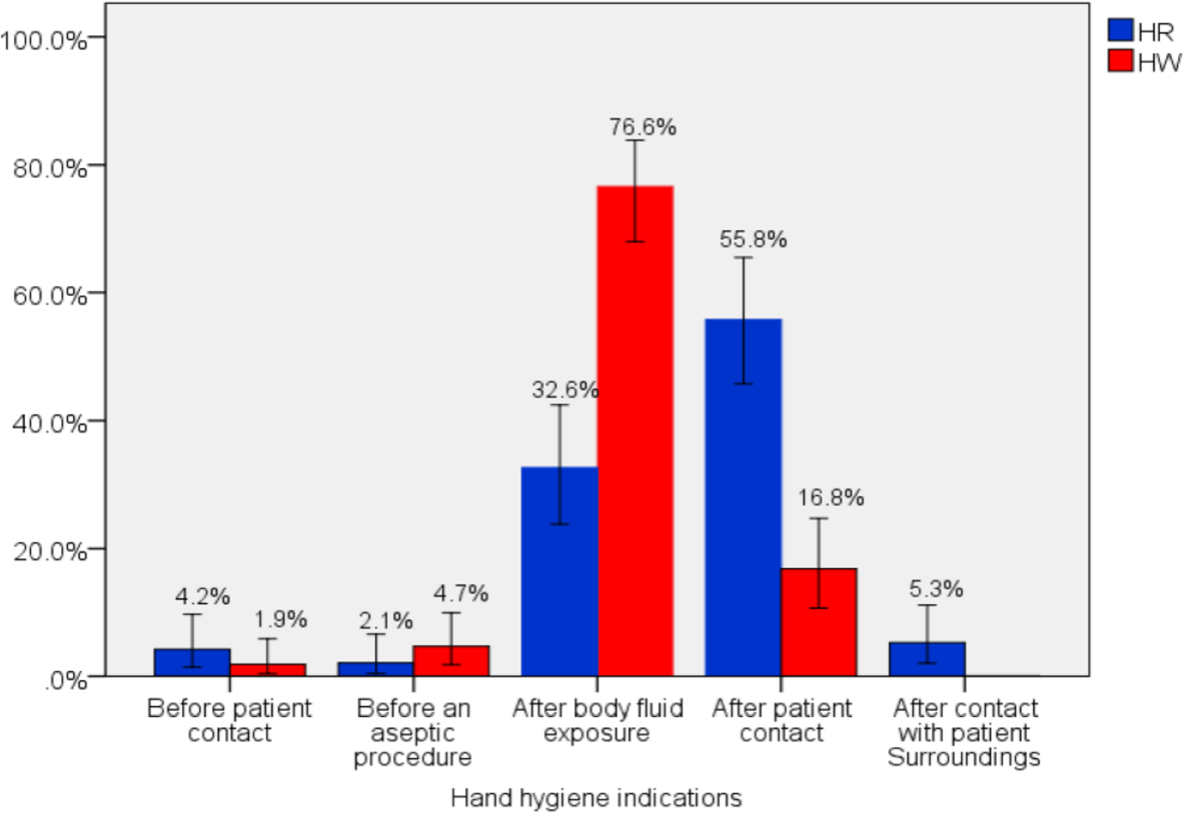
Hand hygiene actions by indications among HCWs in Debre Berhan Referral Hospital, May 2017. HR: Hand rubbing with an alcohol-based handrub; HW: Handwashing with soap and water; Error bars show 95% confidence intervals around hand hygiene action; HCWs: Health care workers

Hand rubbing was performed in 95, (47.0%; 95% CI: 40.2–53.9), out of the 202 hand hygiene actions. Hand rubbing was frequently performed, (55.8%; 95% CI: 45.7–65.5), after patient contact while handwashing with soap and water was more frequent, (76.6%; 95% CI: 67.9–83.9), after body fluid exposure compared with other indications (Fig. 1).

### Hand hygiene resources

In this study, sink to patient beds ratio was 1:4.9, and soap was available to 36.4% of the sinks. Alcohol-based handrub was available for 16.8% (18/107) of the patient beds.

## Discussion

This study captured hand hygiene compliance of HCWs over a 24 h period. Overall hand hygiene compliance was low (22%). HH compliance was low across all professional categories and similar by shift. In line with our study, HH compliance was found low in previous studies [14, 22, 24]. In low-income and middle-income countries, HH compliance was averaged 22·4% before multimodal intervention [11]. Hand hygiene compliance was much lower in the present study compared to post- multimodal intervention studies from India (82%) [15], Kuwait (61.4%) [25], and Colombia (77%) [26]. The possible reason for low compliance in our study might be due to the WHO’s multimodal HH improvement strategy which was not implemented. For instance, HH resources were deficient at the point of patient care. There were no visual reminders for hand hygiene at work place. Similarly, there was lack of HH monitoring and provision of performance feedback to HCWs. Studies have demonstrated that implementation of a multimodal strategy is globally accepted as best approach to achieve HH compliance in health-care settings [11, 27, 28].

Hand hygiene compliance was inconsistent by the five indications for hand hygiene which might be another reason for low compliance. Lower levels of compliance were witnessed for indications before patient contact, before an aseptic procedure and after contact with patient surroundings. By contrast, compliance with hand hygiene was relatively higher after body fluid exposure followed by after patient contact. This suggests that HCWs more likely to perform HH for the indications that protect themselves from microbial contamination and infection rather than that protect patients. Self-protection tendency of HCWs has been identified in multiple studies [11, 25, 29–31].

Hand rubbing is recommended as the gold standard for hand hygiene according to the “my five moments for hand hygiene” in clinical situations [9, 21]. Particularly in resource-constrained settings, the use of alcohol based hand rubs is a practical solution to overcome constraints because they can be distributed individually to staff for pocket carriage and placed at the point of care [20, 21]. In contrary to findings from other studies [11, 12, 32], the present study revealed that hand rubbing was not the preferred means for hand hygiene. One reason could be that alcohol based hand rub was deficient at the point of care and was obstacle to performing HH according to recommendation. Ensuring availability of alcohol-based hand rubs at the point of patient care is a key factor for hand hygiene improvement in previous studies [30, 33, 34].

The strength of this study is that observation was done over a 24 h to minimize selection bias. In addition to this, the HCWs were unaware of being observed to minimize “Hawthorne effect”. This study was not free of limitations. This study solely employed direct observation method. As a result, did not address why HH compliance was found to be low. The cross-sectional results of this study might not be representative of HH compliance throughout the year. This study conducted in a single hospital, and hence the generalizability of our results to other settings might be limited.

## Conclusion

This study showed that HH compliance of HCWs was found to be low. Indications that are high risk to the patient have lower compliance. This suggests that the need of HH compliance improvement strategy is evident. Implementing WHO’s multimodal strategy is crucial to improve HH compliance of HCWs. Access to HH resources should be emphasised as an integral part of HH improvement strategy.

## Additional files

Additional file 1: Hand hygiene compliance observation form. (DOC 407 kb)

Additional file 2: Hand hygiene compliance data set. (SAV 71 kb)

## Abbreviations

CI: Confidence interval
HAI: Health-care associated infection
HCW: Health care worker
HH: Hand hygiene
HHA: Hand hygiene action
HHO: Hand hygiene opportunity
HR: Hand rubbing
HW: Hand washing
NICU: Neonatal intensive care unit
OB/GYN: Obstetrics and gynecology
PSG: Patient safety goal
WHO: World Health Organization
