# Supplementary material for: A twenty-four-hour observational study of hand hygiene compliance among health-care workers in Debre Berhan referral hospital, Ethiopia
Source: Antimicrob Resist Infect Control. 2017 Oct 30;6:109. doi: 10.1186/s13756-017-0268-y (PMC5663127; doi:10.1186/s13756-017-0268-y)
Supplement: Supplementary file 1 — Hand hygiene compliance observation form. (DOC 407 kb) [file 13756_2017_268_MOESM1_ESM.doc]

| | Facility |  | Ward |  | Department |  | | --- | --- | --- | --- | --- | --- | |  | | | | | | | Date: (dd/mm/yy) | / / | Start/End time: (hh:mm) | : / : | Observer:(initials) |  | |  | | | | | | | | | | | | | | | | | |
| --- | --- | --- | --- | --- | --- | --- | --- | --- | --- | --- | --- | --- | --- | --- | --- | --- | --- | --- | --- | --- | --- | --- | --- | --- | --- | --- | --- | --- | --- | --- | --- | --- | --- | --- | --- |
| Prof.cat |  | | Prof.cat |  | | Prof.cat |  | | Prof.cat |  | |
| Code |  | | Code |  | | Code |  | | Code |  | |
| Ses N° |  | | N° |  | | N° |  | | N° |  | |
| Opp. | Indication | HH action | Opp. | Indication | HH action | Opp. | Indication | HH action | Opp. | Indication | HH action |
| 1 |  |  | 1 |  |  | 1 |  |  | 1 |  |  |
|  | | | | | | | | | | | |
| Opp. | Indication | HH action | Opp. | Indication | HH action | Opp. | Indication | HH action | Opp. | Indication | HH action |
| 2 |  |  | 2 |  |  | 2 |  |  | 2 |  |  |

| Opp. | Indication | HH action | Opp. | Indication | HH action | Opp. | Indication | HH action | Opp. | Indication | HH action |
| --- | --- | --- | --- | --- | --- | --- | --- | --- | --- | --- | --- |
| 3 |  |  | 3 |  |  | 3 |  |  | 3 |  |  |

| Opp. | Indication | HH action | Opp. | Indication | HH action | Opp. | Indication | HH action | Opp. | Indication | HH action |
| --- | --- | --- | --- | --- | --- | --- | --- | --- | --- | --- | --- |
| 4 |  |  | 4 |  |  | 4 |  |  | 4 |  |  |

| Opp. | Indication | HH action | Opp. | Indication | HH action | Opp. | Indication | HH action | Opp. | Indication | HH action |
| --- | --- | --- | --- | --- | --- | --- | --- | --- | --- | --- | --- |
| 5 |  |  | 5 |  |  | 5 |  |  | 5 |  |  |

| Opp. | Indication | HH action | Opp. | Indication | HH action | Opp. | Indication | HH action | Opp. | Indication | HH action |
| --- | --- | --- | --- | --- | --- | --- | --- | --- | --- | --- | --- |
| 6 |  |  | 6 |  |  | 6 |  |  | 6 |  |  |

| Opp. | Indication | HH action | Opp. | Indication | HH action | Opp. | Indication | HH action | Opp. | Indication | HH action |
| --- | --- | --- | --- | --- | --- | --- | --- | --- | --- | --- | --- |
| 7 |  |  | 7 |  |  | 7 |  |  | 7 |  |  |

| Opp. | Indication | HH action | Opp. | Indication | HH action | Opp. | Indication | HH action | Opp. | Indication | HH action |
| --- | --- | --- | --- | --- | --- | --- | --- | --- | --- | --- | --- |
| 8 |  |  | 5 |  |  | 8 |  |  | 8 |  |  |

| Opp. | Indication | HH action | Opp. | Indication | HH action | Opp. | Indication | HH action | Opp. | Indication | HH action |
| --- | --- | --- | --- | --- | --- | --- | --- | --- | --- | --- | --- |
| 9 |  |  | 9 |  |  | 9 |  |  | 9 |  |  |

| Opp. | Indication | HH action | Opp. | Indication | HH action | Opp. | Indication | HH action | Opp. | Indication | HH action |
| --- | --- | --- | --- | --- | --- | --- | --- | --- | --- | --- | --- |
| 10 |  |  | 10 |  |  | 10 |  |  | 10 |  |  |

| Opp. | Indication | HH action | Opp. | Indication | HH action | Opp. | Indication | HH action | Opp. | Indication | HH action |
| --- | --- | --- | --- | --- | --- | --- | --- | --- | --- | --- | --- |
| 11 |  |  | 11 |  |  | 11 |  |  | 11 |  |  |
